# Evaluation of Microbial Adhesion and Biofilm Formation on Nano-Structured and Nano-Coated Ortho-Prosthetic Materials by a Dynamic Model

**DOI:** 10.3390/ijerph17031013

**Published:** 2020-02-05

**Authors:** Simone Leonetti, Benedetta Tuvo, Beatrice Campanella, Stefano Legnaioli, Massimo Onor, Emilia Bramanti, Michele Totaro, Angelo Baggiani, Serena Giorgi, Gaetano Pierpaolo Privitera, Nicola Piolanti, Paolo Domenico Parchi, Beatrice Casini

**Affiliations:** 1 Department of Translational Research, N.T.M.S., University of Pisa, via San Zeno, 37/39-56127 Pisa, Italy; s.leonetti@live.com (S.L.); tuvobenedetta@hotmail.it (B.T.); michele.totaro.unipi@hotmail.com (M.T.); angelo.baggiani@med.unipi.it (A.B.); giorgiserena@yahoo.it (S.G.); gaetano.privitera@med.unipi.it (G.P.P.);; 2 Institute of Chemistry of Organometallic Compounds, National Research Council, via Giuseppe Moruzzi, 1-56124 Pisa, Italy; beatrice.campanella@pi.iccom.cnr.it (B.C.); s.legnaioli@pi.iccom.cnr.it (S.L.); massimo.onor@pi.iccom.cnr.it (M.O.); emilia.bramanti@pi.iccom.cnr.it (E.B.); 3 Orthopaedic and Traumatology Division, Azienda ospedaliera-Universitaria Pisana, via Roma, 67-56126 Pisa, Italy; nicpio@hotmail.it

**Keywords:** biofilm, Drip Flow Biofilm Reactor, implantable device-related infections, titanium and cobalt-chrome nano-structured materials, silver nanoparticles, Quorum Sensing

## Abstract

The bio-engineering technologies of medical devices through nano-structuring and coating was recently proposed to improve biocompatibility and to reduce microbial adhesion in the prevention of implantable device-related infections. Our aim was to evaluate the ability of new nano-structured and coated materials to prevent the adhesion and biofilm formation, according to the American Standard Test Method ASTM-E2647-13. The materials composition was determined by X-ray Fluorescence and Laser Induced Breakdown Spectroscopy. Silver release was evaluated by Inductively Coupled Plasma Mass Spectrometry analysis. The gene expression levels of the Quorum Sensing *Las* and *Rhl* system were evaluated by the ΔΔCt method. The Log bacterial density (Log CFU/cm^2^) on TiAl6V4 was 4.41 ± 0.76 and 4.63 ± 1.01 on TiAl6V4-AgNPs compared to 2.57 ± 0.70 on CoCr and 2.73 ± 0.61 on CoCr-AgNPs (P < 0.0001, A.N.O.V.A.- one way test). The silver release was found to be equal to 17.8 ± 0.2 µg/L after the batch phase and 1.3 ± 0.1 µg/L during continuous flow. The *rhlR* gene resulted in a 2.70-fold increased expression in biofilm growth on the silver nanoparticles (AgNPs) coating. In conclusion, CoCr showed a greater ability to reduce microbial adhesion, independently of the AgNPs coating. The silver release resulted in promoting the up-regulation of the *Rhl* system. Further investigation should be conducted to optimize the effectiveness of the coating.

## 1. Introduction

Even if the total joint replacement is among the most successful surgical procedures in terms of consistent improvement of the patient’s quality of life, it may be not free of complications, and infections are among the most challenging ones. Infections related to the implant of orthopedic devices are included within the description of surgical site infections (SSI), and they have great relevance for the significant clinical and financial impact on the patients, as well as for the treating surgeon and the healthcare system [1]. Despite the progress in diagnostics in recent years, the real percentage of peri-prothesis infection (PPI) is probably underestimated. Barberan J. et al. [2], reported that surgical site infections represent a potential complication in prosthetic orthopedic surgery, with an incidence between 0.5% and 3% for the first total hip replacement and total knee replacement implants, reaching up to 20% in case of revisions. Moreover, the PPI is the third cause of prosthetic implant failure in primary surgery and is the leading cause in revision surgery within the first five years [3]. More recently, these data have been confirmed by Kenney C. et al. in a systematic review of the causes of failure of revision total hip arthroplasty [4].

The bio-engineering technologies of medical devices through nano-structuring and coating has recently been proposed, not only to prevent microbial adhesion, but also to improve the biocompatibility of materials [5,6,7,8] to develop the ideal orthopedic implant.

Conversely, in the Second International Consensus Meeting on Prosthetic Joint Infection, it was stressed that the duration of long-term anti-infective effect of the modified implant surface is unknown, and that further studies are needed [9].

Titanium (Ti6Al4V) and cobalt chromium (CoCr) implants are widely used in the orthopedic field due to their mechanical and biological proprieties [10,11]. Among the nano-coating technologies, silver nanoparticles (AgNPs) are the most used for their bactericidal and cytotoxic activity on microorganisms [12,13], but their instability is often underestimated. The different values of pH and oxygen concentrations can cause AgNPs’ dissociation or aggregation, reducing their stability and their adhesion on surfaces [14].

The efficacy of nano-structuring and coating materials should be evaluated through appropriate experimental models [15]. Static models are particularly useful and easy to set up for examining early events in bacterial biofilm formation and to identify signals that modulate the transition from a planktonic to a biofilm mode of growth. Because most of the cultures are neither continuously supplied with fresh medium nor are they aerated, there may be a limitation of nutrients, which may encourage an inability to easily generate mature biofilms. The static models generate a stable structure and limits the possibility of cell detachment, ensuring that observed differences in cell numbers are due to cell death rather than detachment [16]. The dynamic models in continuous flow enable the formation of mature biofilms with optimal conditions mimicking real conditions in vitro [16,17]. Among the advantages of continuous-flow models is the ability to compare the effects that different media, oxygen concentrations, temperature shifts, and substances exert on a biofilm at any developmental phase. These models also allow for evaluating the effects that transiently occurring molecules, such as antibiotics or adherence inhibitors, have on biofilms. However, the technical disadvantages of continuous-flow biofilms include increased experimental complexity as well as possible formation/trapping of air bubbles in the setup tubing [15].

The aim of this study is to evaluate the ability of new titanium and cobalt-chrome nanostructured materials, coated or not with AgNPs, to prevent and reduce the adhesion and microbial biofilm formation. This evaluation was conducted according to the standard ASTM-E2647-13, the standard test method for quantification of a *Pseudomonas aeruginosa* biofilm grown using a Drip Flow Biofilm Reactor with low shear and continuous flow. The release of AgNPs and its effect on the Quorum Sensing genes regulation were also determined.

## 2. Methods

### 2.1. Coupon Materials

Coupons with a surface of 18.75 cm^2^ (25 × 75 × 1 mm) were made in nanostructured titanium (TiAl6V4) and cobalt-chrome, with or without AgNPs coating. A total of 32 coupons were tested (TiAl6V4 n = 8, TiAl6V4-Ag n = 8, CoCr n = 8, CoCr-Ag n = 8). The number of AgNPs used for coating was unknown because this was covered by industrial property rights, though it was experimentally assessed determined by X-ray Fluorescence (XRF) and Laser Induced Breakdown Spectroscopy (LIBS).

### 2.2. The Drip Flow Biofilm Reactor (DFBR)

For the cell adhesion capacity analysis, biofilms were grown on the coupons in four parallel flow chambers DFBR (four-chamber Drip Flow Biofilm Reactor^®^, Biosurface Technologies Corporation, Bozeman, MT, USA). Each channel had an individual lid fixed with screws that kept the conditions aseptic during the sampling process. Each channel contained a coupon of 18.75 cm^2^. This bioreactor is recommended by the ASTM Standard Method E2647-13 [18] for growing, sampling and analyzing a *Pseudomonas aeruginosa* biofilm formed under low shear and continuous flow, trying to mimic the environmental conditions found in medical devices and the human body [16].

### 2.3. Culture and Inoculum Preparation

An isolated colony of *Pseudomonas aeruginosa* (ATCC 700888) from an R2A plate (VWR chemicals, Radnor, PA, USA) was aseptically removed and inoculated into 100 mL of sterile Tryptic Soy Broth 3 gr/L (TSB, Merck Millipore, Burlington, MA, USA) and the bacterial suspension was incubated in an environmental shaker at 35 ± 2°C for 20 to 24 h. The viable bacterial density was evaluated equal to 10^8^ CFU/mL by serial dilution and plating.

### 2.4. DFBR Batch Phase

After setting up the reactor as described in the user manual, each coupon was inserted into each reactor channel (4 channels), and 15 mL of sterile TSB 3 gr/L and 1 mL of bacterial inoculum were aseptically added in each channel. The reactor system was incubated at room temperature (21 ± 2°C) for 6 h, in the level position.

### 2.5. Continuous Flow Phase

After the static phase, the influent nutrient tubing line was aseptically connected to the carboy containing the continuous flow nutrient broth (sterile TSB 270 mg/L), each line was attached through a pump head and a sterile needle was connected at the end of each line. The continuous flow of nutrients was pumped into the reactor through a pump set at a flow rate equal to 200 mL/h (50 mL/h per channel). The reactor was operated in continuous flow mode for 48 h.

### 2.6. Biofilm Sampling and Population Density Determination

After 48 h, each coupon was aseptically removed from the channel and it was immersed in 45 mL of sterile buffered water (0.0425 g/L KH_2_PO_4_ distilled water, filter sterilized and 0.405 g/L MgCl·6H_2_O distilled water, filter sterilized). The biofilm-covered coupon surface was scraped in a downward direction for approximately 15 s, using the flat end of a sterile spatula. The scraping process was repeated 3 to 4 times, throughout the entire surface of the coupon. A total of 1 mL of sterile buffered water was pipetted over the top surface of the coupon for a total of 5 rinses. The final volume was 50 mL.

The scraped biofilm samples were homogenized at 20,500 ± 5000 r/min for 30 s. The homogenizer was decontaminated before next use with 70% ethanol for 15 s and rinsing with sterile buffered water for 30 s. Total bacterial count was assessed for each sample by serial 10-fold dilutions and plating each dilution in duplicate in R2A (VWR chemicals, Radnor, PA, USA), incubated for 17 to 20 h at 35 ± 2 °C. Biofilm population density was recorded as Log colony forming units on surface area.

### 2.7. Statistical Analysis

Analysis of Variance, ANOVA, One-way test and Holm-Sidak’s multiple comparisons test were performed to observe statistical significance between coupons. The statistic elaboration was conducted by GraphPad Prism^®^ version 8.0.1 (GraphPad Software Inc., San Diego, CA, USA).

### 2.8. Coupons Analysis and Silver Release

Each type of coupon was tested by XRF and LIBS analyses.

X-ray Fluorescence was performed using the Elio ED-XRF portable spectrometer (XGLab, Italy). The X-ray tube had a Rh anode and the measurements area was about 1 mm^2^. The spectrometer resolution was 130 eV at the Mn Kα line. The acquisition time, energy of the X-ray tube and current were set at 90 s, 40 keV and 40 μA, respectively.

LIBS analyses were done using Modì (MARWAN TECHNOLOGY, Pisa, Italy), a mobile LIBS instrument equipped with a dual pulse laser (Nd-YAG, λ = 1064 nm, single pulse energy up to 80 mJ in 100 ns) and a non-intensified double grating spectrometer (AvaSpec Dual-Channel Fiber Optic Spectrometer from Avantes). The spectrometer simultaneously covered the spectral interval between 200 and 430 nm (with a resolution of 0.1 nm) and between 415 and 900 nm (with resolution of 0.3 nm).

Silver release was assessed in the culturing medium, during the batch phase (6 h later) and during the continuous flow after 18-30-42-54 h. A negative control, through the medium without coupons contact, was carried out at time zero from the beginning of the test.

An Agilent 7700x inductively coupled plasma mass spectrometer (ICP-MS) (Agilent Technologies, Santa Clara, CA, USA) equipped with a MicroMist nebulizer and a Peltier cooled (2 °C) quartz Scott-type double pass spray chamber was used for the quantification of ^107^Ag and ^109^Ag. A solution of 10 μg/L iridium in 2% HNO_3_ was used as internal standard. The operating parameters for ICP-MS were optimized with a tuning solution containing 1.0 ng/mL of Ce, Co, Li, Mg, Tl and Y in 2% HNO_3_. Silver quantitation was based on external calibration with standard solutions of AgNO_3_ (6 solutions in the 0.5–20 ng/mL range). Quality control was performed by analyzing a 5 ng/mL AgNO_3_ standard after each sample. For the analysis, 2 mL of sampled TSB were treated with 2 mL of 69% HNO_3_ and maintained at 90°C for 1 h. Subsequently 2 mL of 30% H_2_O_2_ were added and the sample placed again at 90 °C for 1 h. Before the analysis, the sample was diluted 1:10 in deionized water.

### 2.9. Quorum Sensing Expression Analysis

The effects of silver on the QS genes expression of *P. aeruginosa* growth on TiAl6V4-Ag coupons, were evaluated with mRNA extraction according to the RNeasy PowerBiofilm protocol (Qiagen, Germantown, MD, USA), determination of RNA concentration was through using a spectrophotometer, reverse transcription was according to the QuantiNova Reverse Transcription protocol (Qiagen, USA), and finally amplification of the target genes by realtime-PCR according to the QuantiNova SYBR Green PCR protocol (Qiagen, USA). The target genes were *lasR* and *rhlR* whilst *rpoD* was considered the housekeeping gene. The primers used for the PCR reaction were for *lasR* forward: 5′-AAGGAAGTGTTGCAGTGGTG-3′, reverse: 5′-GAGCAGTTGCAGATAACCGA-3′; for *rhlR* forward: 5′-GACCAGGAGTTCGACCAGTT-3′, reverse: 5′-GGTAGGCGAAGACTTCCTTG-3′; for *rpoD* forward: 5′-CTGCAGGCCCTGGGCGAGAA-3′, reverse: 5′-CTCGGGCGATCAACTCTTTC-3′ [19].

The thermal cycling protocol, carried out using the Bio-Rad CFX Connect Real-Time PCR Detection System (Bio-Rad Laboratories, Berkeley, CA, USA), was: 95 °C, 15 min; 35 cycles of (95 °C, 30 s; 60 °C, 30 s; 72 °C, 30 s); final extension at 72 ° C for 10 min and 4 °C hold. Data analysis was carried out using the Bio-Rad CFX Manager 3.1 version 3.1.1.5.17.0823 program (Bio-Rad Laboratories, Berkeley, CA, USA) and the analysis of the relative expression was conducted using the Livak equation (2^−ΔΔ*CT*^) [20].

## 3. Results

### 3.1. Population Density Determination

**Figure 1:**
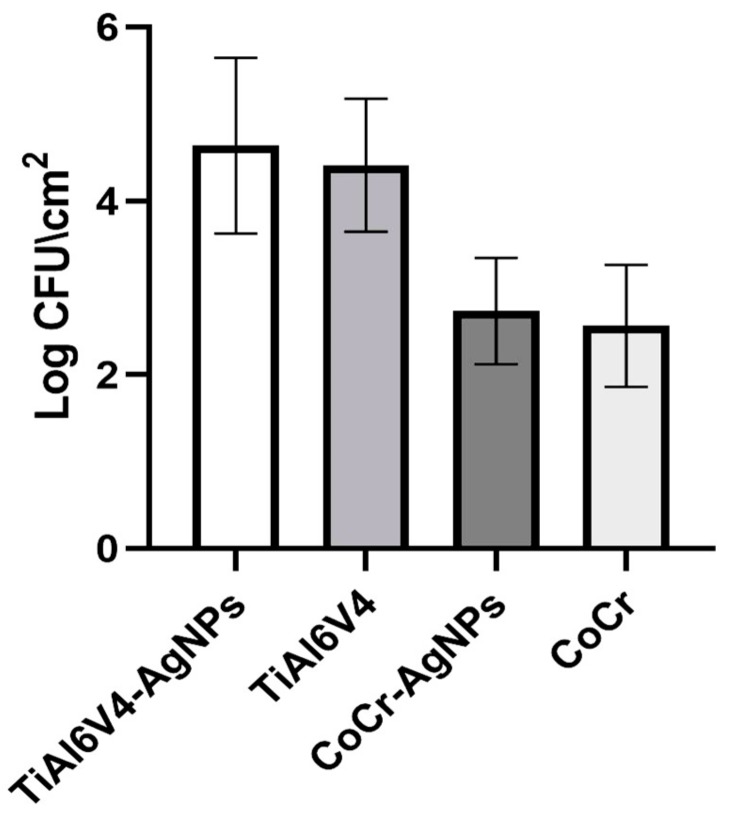
Log population density (means and standard deviations) for each material evaluated.

The results of the population density determination, obtained by analyzing eight coupons by the type of material, showed a Log bacterial density (Log CFU/cm^2^) equal to 4.41 ± 0.76 on TiAl6V4 coupons and 4.63 ± 1.01 on TiAl6V4-AgNPs coupons. Lower values were found on CoCr coupons, respectively equal to 2.57 ± 0.70 on CoCr and 2.73 ± 0.61 on CoCr-AgNPs (Figure 1). This difference was statistically significant. The P-value obtained by ANOVA one-way test was <0.0001, and results obtained by Holm-Sidak’s multiple comparisons test were significant between titanium and chromium-cobalt (<0.0001), but not significant between the same material coated and not coated (>0.92).

### 3.2. Coupons Analysis and Silver Release

**Figure 2:**
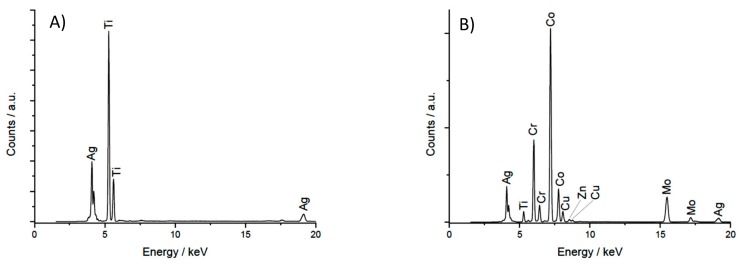
X-ray Fluorescence (XRF) spectra of TiAl6V4-AgNP (**A**) and CoCr-AgNP (**B**) coupons.

The XRF results are reported in Figure 2.

XRF spectra of TiAl6V4 coupons coated with AgNPs confirmed the presence of both Ti and Ag on the coupons’ surface, while the spectra from CoCr-AgNPs showed copper as well as chrome, cobalt and silver.

Multiple acquisitions on the same point by LIBS allowed for collecting information from successive layers of the coupon, estimating 1–2 µm as the depth of the laser pulse [21].

**Figure 3:**
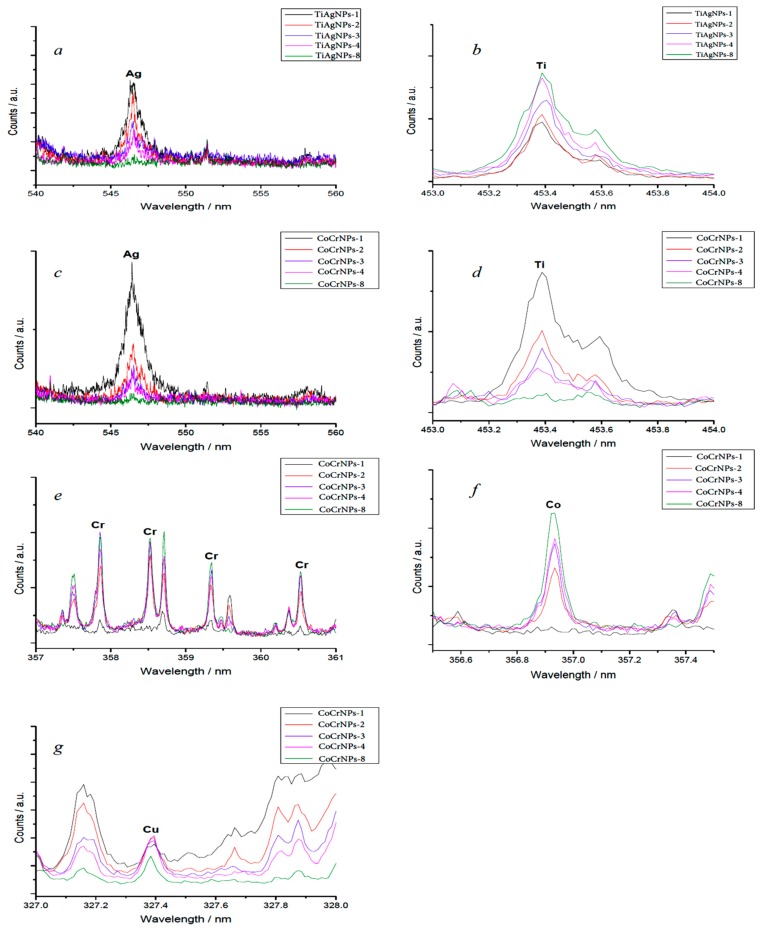
Insets of Laser Induced Breakdown Spectroscopy (LIBS) spectra relative to Ag (**a**) and Ti (**b**) in TiAl6V4-AgNPs coupons and Ag (**c**), Ti (**d**), Cr (**e**), Co (**f**), Cu (**g**) in CoCr-AgNPs coupons. Sample numbering indicates the progressive order by which the analysis was made by repeatedly shooting the laser on the same point.

In-depth LIBS measurements revealed that in both types of coupons silver was present, mostly in the first 1–2 µm layers. After 8 laser shots on the same point, the signal from Ag was no longer detectable. Titanium, conversely, was present homogeneously throughout the TiAl6V4-AgNPs coupons. On the surface of CoCr-AgNPs coupons, besides silver, titanium was also detectable, which may be a pollutant from the manufacturing process. Chromium, cobalt and copper were homogeneous throughout the coupons (Figure 3).

**Figure 4:**
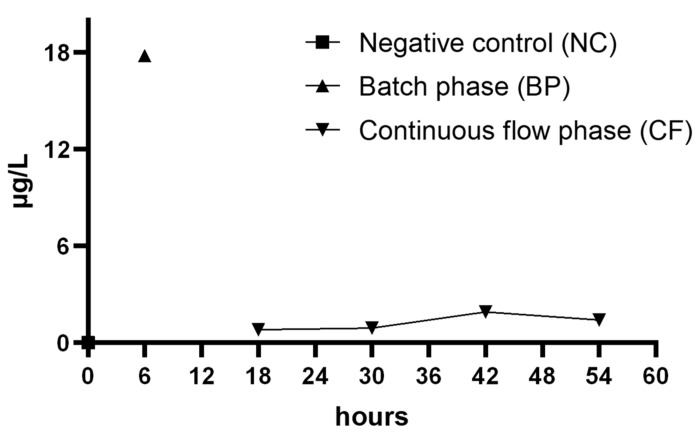
AgNPs release in the culturing medium during all phases of biofilm growth.

The AgNPs’ release in culturing medium was evaluated by ICP-MS analysis (Figure 4). The concentration of Ag, corrected for dilutions, was equal to 17.8 ± 0.2 µg/L during the batch phase. During the continuous flow, the concentration for each 12 h following the batch phase were 0.9 ± 0.1 µg/L; 0.8 ± 0.1 µg/L; 1.9 ± 0.2 µg/L and 1.4 ± 0.1 µg/L respectively. The negative control was free from AgNPs.

### 3.3. Quorum Sensing Expression Analysis

Results obtained by the relative expression analysis conducted by the Livak equation showed a 2.70-fold change of the *rhlR* gene expression in cell growth in the presence of AgNPs (TiAl6V4-AgNPs vs. TiAl6V4) and a 0.33-fold change of the *lasR* gene.

## 4. Discussion

The results obtained in this study showed that AgNPs were released by the coated coupons and the remaining amount on the surface was unable to reduce microbial adhesion. In fact, no statistically significant difference was observed between coated and noncoated materials, regardless of their alloy. CoCr coupons demonstrated better properties for reducing the biofilm attachment.

Clinical studies showed the release of nanoparticles and ions due to the corrosion of orthopedic implants, causing significant production of inflammatory cytokines by tissues [22,23]. The toxicity of AgNPs was studied by AshaRani et al. on human lung fibroblast cells and human glioblastoma cells, demonstrating a cytotoxic, genotoxic, and antiproliferative rule of AgNPs at concentrations >100 μg/mL. [24].

The release of the silver coating is associated with the pH value of the medium, which modifies the surface charge and promotes the oxidative dissolution of the AgNPs, as well as the formation of aggregates by particles. The behavior of the particles varies in acid and alkaline conditions; the size of the particle aggregates decreases with increasing pH, but it increases the stability, as reported by Fernando et al. [14]. The inhibitory effect of silver on microbial cells was evaluated; a study conducted by Yen-Chi Chen et al. [25] reported that 13.48 µg/mL (0.01348 µg/L) was the minimum AgNP concentration needed to inhibit the growth of *Escherichia coli*. Gholamrezazadeh et al. [26] reported that 20.0 ± 0.2 µg/L AgNPs Minimum Inhibitory Concentration (MIC) were capable of inhibiting *P. aeruginosa* growth. In our study, after 6 h of batch phase, the release of AgNPs was equal to 17.8 ± 0.2 µg/L, a concentration unable to prevent the planktonic cells attachment of *P. aeruginosa* on the surface. Regarding the effects of silver nanoparticles on the QS biofilm of *P. aeruginosa*, our results confirmed a study conducted by M. Gholamrezazadeh et al. [26], wherein they reported that AgNPs doubled the increase of the expression of the *rhlR* gene. Conversely, the *lasR* gene was more expressed in biofilm growth on the noncoated titanium coupons. In this stage of our study, gene expression analysis was performed only on biofilms grown on titanium coupons, but further research will need to be conducted to verify if this result is also obtained on cobalt-chromium coupons. In any case, the increased expression of the investigated genes is associated with the presence of silver and this could occur independently of the support material.

Las is often considered the most important system in the P. aeruginosa QS regulation, since it is required to activate many genes including those coding for Rhl system. Recently, Kostylev M. et al. [27] report that the Rhl system can be activated in the absence of an active Las. This could explain the results obtained in our study, where a greater expression of the rhlR gene compared to the lasR gene was found. The RhlR Quorum Sensing receptor controls the *P. aeruginosa* expression of genes required for biofilm formation as well as genes encoding for virulence factors [28].

CoCr and titanium nanostructured prostheses are widely used for a combination of favorable properties, including mechanical strength, corrosion resistance and biocompatibility. Patel et al. [29] evaluated *Staphylococcus aureus* biofilm formation on CoCr and titanium alloy spinal implants in static condition, determining the optical density by spectrophotometer, and their results showed that titanium better prevented the biofilm formation then the CoCr alloy spinal implants. Castellanos et al. [30] evaluated *Staphylococcus epidermidis* biofilm formation on metal plates of titanium (Ti), porous titanium (p-Ti), cobalt-chromium (CoCr) and stainless steel (SS) in static condition. Their results showed a low adherence in CoCr without a significant difference compared to titanium plates. Our results, obtained with a different strain and in dynamic conditions, demonstrated that CoCr had a higher capacity to reduce the biofilm growth with a significant difference, as compared to titanium.

Regarding the resistance to mechanical stress, in clinical studies conducted by Smith et al. [31,32] reported an increasing rates of spinal rod fractures increased with the decreasing fatigue strength of the material employed, and an increase in fractures was found with titanium (Ti) rods compared to cobalt chromium (CoCr) rods after posterior spinal arthrodesis. Madl et al. [33] analyzed the toxicological implications of particles generated by the wear of metal-on-metal hip implants made of cobalt-chromium-molybdenum (CoCrMo) alloy, demonstrating a greater bio-persistence of CoCr particles and the induction of the inflammatory response. Although the results of animal studies showed that CoCr wear debris can disseminate into distant organs, there is still not enough evidence on the toxicity of these alloys [34]. Studies conducted by Baldwin et al. [35] highlighted that the inflammatory profile induced by CoCr remained consistent and elevated during the 28-day period with high cell counts associated with the implants and a progressive recruitment of T lymphocytes. Studies conducted by Walker [36] et al. in patients undergoing CoCr unicondylar knee arthroplasty, with a history of metal hypersensitivity, showed that the functional outcome and post-intervention survivorship were equivalent to those reported by patients without a history of metal hypersensitivity. In a systematic review conducted by Christian et al. [37] no evidence was found related to a causative relationship between CoCr-containing hip implants and increased risk of systemic cancers. Further investigations are required to ensure safety in the use of this alloy.

In our study, the XRF and the LIBS analyses allowed us to qualitatively evaluate the coupons surface composition and to demonstrate the quality of production of these alloys. In this context, the CrCo analysis highlighted the pollution by copper and titanium probably due to manufacturing process errors.

Our study is one of the few in which biofilm adhesion was evaluated on implantable medical devices by a standard protocol, which provides for use of a dynamic model in continuous flow with optimal conditions mimicking a real scenario.

Our study had some limitations. It was not possible to compare the results obtained from similar studies that used the same materials and the dynamic study model of the biofilm. Therefore, it is desirable that this type of investigation be carried out in the future through the use of protocols that have verified reproducibility, such as the ASTM standard protocol.

Moreover, the bacterial strain recommended by the ASTM standard is not the most representative of strains associated with implantable device-related infections. Other strains, such as *Staphylococcus epidermidis* or fungal strains should be included and standardized in a new protocol.

Further studies need to be conducted in this field to achieve a deeper understanding of the concentration needed to increase the toxicity of AgNPs on biofilm-associated bacteria, without causing damage to host tissues and to understand how much this concentration is able to regulate the QS genes expression and then control the virulence of these strains.

## 5. Conclusions

The experimental model used allowed us to demonstrate that among the nanostructured materials, the CoCr alloy was the one with the greatest capacity to reduce microbial adhesion and biofilm formation.

The AgNPs nano-coating did not show influence on microbial adhesion, but the concentration used revealed the ability to induce the expression of one of the main QS systems, thus activating the virulence of the microbial strain studied.

The release of AgNPs in the culture medium highlighted the instability of the nano-coating.

In order to prevent implantable device-related infections, it is necessary to improve the ability of the nano-coating to inhibit microbial adhesion.

The inflammatory profile induced by CoCr and the formation of debris by alloy prostheses remain an interesting field of study to ensure safety in the use of these materials.
